# Genetic polymorphism of 19 autosomal STR loci in the Yi ethnic minority of Liangshan Yi autonomous prefecture from Sichuan province in China

**DOI:** 10.1038/s41598-021-95883-x

**Published:** 2021-08-11

**Authors:** Jingliang Cheng, Binghui Song, Jiewen Fu, Xiaoli Zheng, Tao He, Junjiang Fu

**Affiliations:** 1 Key Laboratory of Epigenetics and Oncology, The Research Center for Preclinical Medicine, Southwest Medical University, Luzhou, 646000 Sichuan China; 2 Laboratory of Forensic DNA, The Judicial Authentication Center, Southwest Medical University, Luzhou, 646000 Sichuan China

**Keywords:** Biological techniques, Genetics

## Abstract

The Yi is one of fifty-six ethnic populations and one of the most ancient ethnic groups in China. The Liangshan Yi Autonomous Prefecture (LYAP) in Sichuan Province has the single largest Yi community in China. To establish a Yi population database in the LYAP of Sichuan in China, a Goldeneye™ DNA Identification System 20A Kit with 19 autosomal STRs (short tandem repeats) was used. As a result, the total discrimination power (TDP) and the cumulative probability of exclusion (CPE) for these STRs in 1016 unrelated individuals were 0.999999999999999999999897 and 0.9999999597, respectively. Totals of 273 alleles for 19 STRs and 8–22 alleles for each locus were found. The allelic frequencies ranged from 0.0005 to 0.5084. The forensic parameter averages of these STRs were as follows: observed heterozygosity (H_obs_) of 78.44%, expected heterozygosity (H_exp_) of 79.89%, discrimination power (DP) of 92.66%, and probability of exclusion (PE) of 57.68%. Penta E presented the highest levels of H_obs_ and DP, whereas TPOX showed the lowest H_obs_ and DP values. Nei’s standard genetic distance matrix among 31 populations found that the nearest genetic distance to the Yi population was the Sichuan Han (0.0056). Altogether, we first reported the forensic parameters and allele frequencies of 19 autosomal STRs of the Yi group in Liangshan. These 19 STR makers could provide highly informative polymorphisms for individual identification, paternity testing and genetic population analyses.

## Introduction

The Yi is one of fifty-six ethnic populations and one of the most ancient ethnic groups in China. According to the report of Sixth National Census data in 2010, the Yi is the sixth-largest ethnic minority in China, with a population of 8,714,393. The Liangshan Yi Autonomous Prefecture (LYAP) in Sichuan Province has the single largest Yi community in China with nearly 50% of the 4.5 million inhabitants^1^. The Honghe and Chuxiong regions in Yunnan Province are the second-largest Yi communities in China. Yi individuals from both Sichuan and Yunnan live in Southwest China. In addition, the Yi ethic group has their own culture and language which belongs to the Loloish language and Sino-Tibetan language close to Burmese^2^.

Allele frequencies and population data were reported for 9, 15 and 20 autosomal short tandem repeat (STR) loci in the Yi ethnic minority from Yunnan Province of China in 2006 and 2017, respectively^3–5^. Genetic diversity and phylogenetic characteristics obtained from non-combined DNA index system (CODIS) STR markers in the Yi ethnic minority with 95 individuals from Sichuan Province were reported in 2018^6^. Genetic polymorphism studies of the Yi minority in this LYAP area using the Y-STR amplification system have been intensively investigated^1,6,7^. Moreover, rapidly mutating study for Y-STRs in this Yi population from Sichuan was also reported^8^. In addition, our group evaluated 23 Y-STR locus mutation rates in Chinese Han father-son pairs from Southwest China^9^. Forensic characteristics and phylogenetic analyses for 19 X-chromosomal STR loci in the LYAP Yi minority was also reported^2^. By using insertion/deletion markers, forensic features and population genetic structure of Dong, Yi, Han, and Chuanqing populations in Guizhou of Southwest China were recently determined^10^. Due to the rich history and complex ethnic makeups in China, it is necessary to study allele frequency for autosomal STRs in different geographic areas and different ethnic groups including Yi Chinese in the LYAP. However, the genetic polymorphisms for autosomal STR loci of the Yi minority in the LYAP are unclear, and the genetic relationships of populations between the Yi minority and other populations are unknown. It is necessary to use autosomal STRs to fill the gap among Y-STRs and X-STRs of this Yi minority. Thus, in this study we focus on establishing a Yi population database from the LYAP in China by using the Goldeneye™ DNA Identification System 20A Kit, which includes 19 autosomal STRs.

## Results

### Linkage disequilibrium, forensic parameters and allele frequencies

We first performed a linkage disequilibrium (LD) analysis of 19 autosomal STR loci in the Yi group in LYAP. For the LD test, only 3 pairs (Supplementary Table S1) of loci showed significant LD after Bonferroni correction (p = 0.05/171 ≈ 0.0003), indicating that most loci were statistically independent. These few pairs in linkage disequilibrium may be due to random sampling errors because the pairs were located on different autosomal chromosomes or chromosome arms.

The forensic parameters, allelic frequencies and p-values of exact tests for Hardy–Weinberg equilibrium (HWE) of 19 autosomal STR loci in the Yi group in Liangshan are presented in Table 1 and Supplementary Table S2. In the present study, eighteen of the 19 STR loci were observed to show HWE after Bonferroni correction (p = 0.05/19 ≈ 0.0026), and only the D21S11 locus was a significant HWE departure. Individual migration or genetic exchange may be a possible explanation for this deviation. The total discrimination power (TDP) and the cumulative probability of exclusion (CPE) of the 19 STRs in 1016 unrelated individuals were 0.999999999999999999999897 and 0.9999999597, respectively.

**Table 1:** The corresponding forensic statistical parameters for 19 autosomal STR loci of the Yi ethnic minority from Liangshan Yi autonomous prefecture in Sichuan Province, Southwest China (n = 1016).

Loci	Allele number	Genotype number	H_exp_	H_obs_	MP	DP	PIC	PE	TPI	H	h	p
D19S433	18	82	0.8272	0.7992	0.0499	0.9501	0.8071	0.5976	2.4902	0.2008	0.7992	0.0201
D5S818	11	32	0.7774	0.7657	0.0856	0.9144	0.7430	0.5371	2.1345	0.2343	0.7657	0.3863
D21S11	20	75	0.8278	0.7805	0.0490	0.9510	0.8067	0.5634	2.2780	0.2195	0.7805	0.0001
D18S51	18	80	0.8514	0.8297	0.0381	0.9619	0.8336	0.6553	2.9364	0.1703	0.8297	0.0575
D6S1043	22	94	0.8752	0.8455	0.0292	0.9708	0.8612	0.6860	3.2357	0.1545	0.8455	0.0048
D3S1358	14	27	0.7084	0.7136	0.1423	0.8577	0.6555	0.4496	1.7457	0.2864	0.7136	0.6977
D13S317	8	28	0.7957	0.7815	0.0722	0.9278	0.7653	0.5652	2.2883	0.2185	0.7815	0.2765
D7S820	13	30	0.7851	0.7657	0.0773	0.9227	0.7525	0.5371	2.1345	0.2343	0.7657	0.1415
D16S539	10	32	0.7811	0.7943	0.0846	0.9154	0.7460	0.5885	2.4306	0.2057	0.7943	0.2941
CSF1PO	10	30	0.7275	0.7077	0.1151	0.8849	0.6845	0.4402	1.7104	0.2923	0.7077	0.1634
Penta D	13	50	0.7982	0.7776	0.0633	0.9367	0.7742	0.5581	2.2478	0.2224	0.7776	0.1082
vWA	12	33	0.8060	0.7874	0.0669	0.9331	0.7761	0.5759	2.3519	0.2126	0.7874	0.1423
D8S1179	9	39	0.8415	0.8317	0.0460	0.9540	0.8206	0.6591	2.9708	0.1683	0.8317	0.4131
TPOX	9	19	0.6419	0.6230	0.1846	0.8154	0.5860	0.3194	1.3264	0.3770	0.6230	0.2183
Penta E	21	141	0.9073	0.8917	0.0161	0.9839	0.8993	0.7786	4.6182	0.1083	0.8917	0.0980
TH01	11	26	0.6752	0.6703	0.1517	0.8483	0.6286	0.3838	1.5164	0.3297	0.6703	0.7461
D12S391	19	70	0.8339	0.8327	0.0492	0.9508	0.8133	0.6610	2.9882	0.1673	0.8327	0.9469
D2S1338	14	68	0.8513	0.8553	0.0410	0.9590	0.8338	0.7054	3.4558	0.1447	0.8553	0.6922
FGA	21	93	0.8668	0.8504	0.0323	0.9677	0.8521	0.6973	3.3609	0.1496	0.8504	0.1588

*H*_*exp*_ expected heterozygosity, *H*_*obs*_ observed heterozygosity, *MP* matching probability, *DP* discrimination power, *PIC* polymorphism information content, *PE* probability of exclusion, *TPI* typical paternity index, *H* Homozygotes, *h* Heterozygotes, *p* p value of the exact test in Hardy–Weninbegy equilibrium.

A total of 273 alleles for 19 STRs with corresponding allelic frequencies from 0.0005 to 0.5084 were noticed, and 8–22 alleles for each locus were found (Supplementary Table S2). The forensic parameter averages of these STRs were as follows: observed heterozygosity (H_obs_) of 78.44%, expected heterozygosity (H_exp_) of 79.89%, discrimination power (DP) of 92.66%, and probability of exclusion (PE) of 57.68%. Locus Penta E presented the highest levels of H_obs_ (0.8917) and DP (0.9839), whereas TPOX showed the lowest H_obs_ of 0.6230 and DP of 0.8154.

### Population comparisons and population differentiation

**Figure 1:**
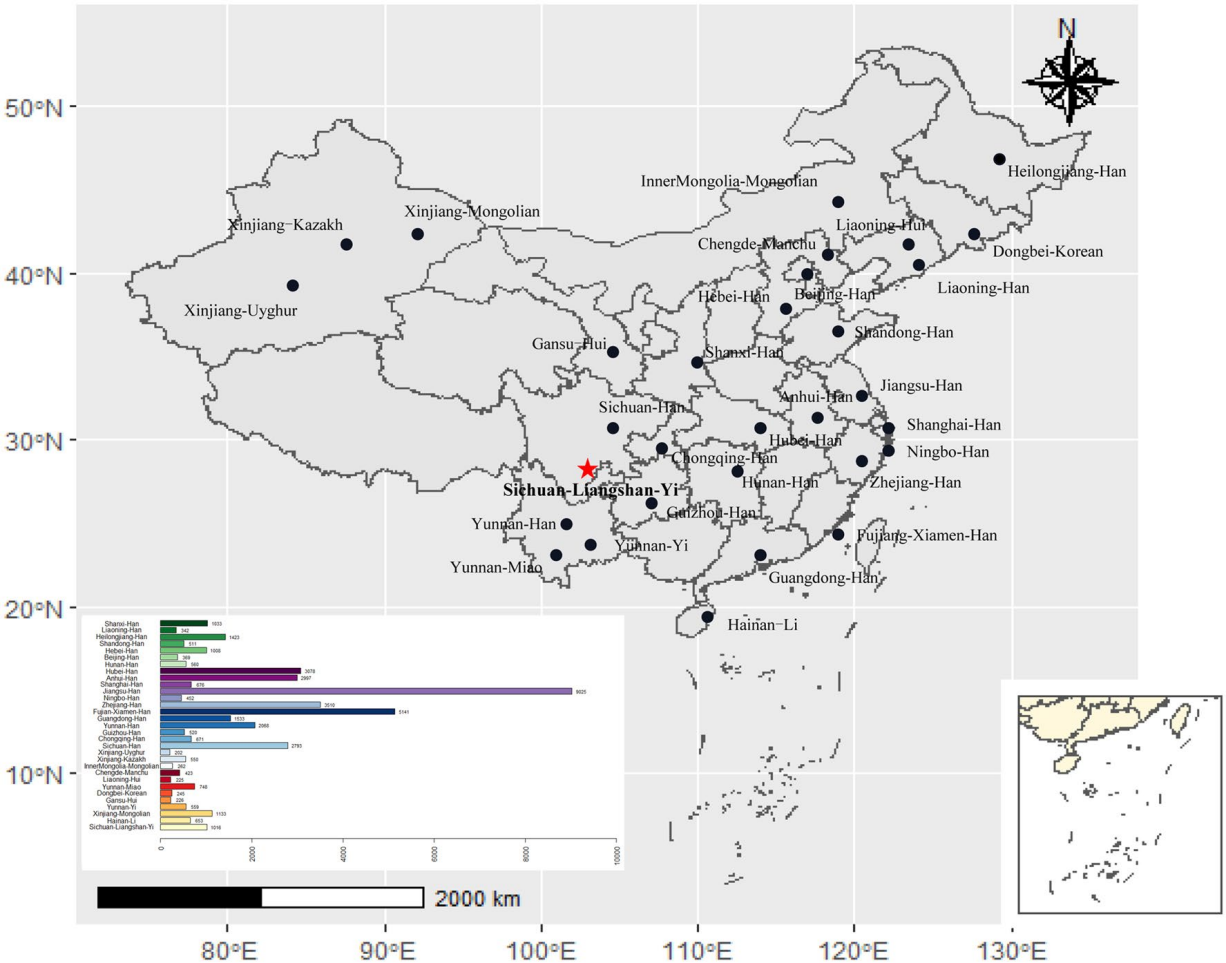
Map showing the geographic positions of the studied Yi ethnic minority in Liangshan Yi Autonomous Prefecture of Sichuan and the 30 reference populations. The sample numbers are presented at the bottom left of the figure. The longitude and latitude of all 31 reference populations are presented in Supplementary Table S3. The R project software (version 4.0.4) (https://www.r-project.org/) and DATAV GeoAtlas (areas_v3) (https://datav.aliyun.com/tools/atlas/index.html) were used to create this map.

**Figure 2:**
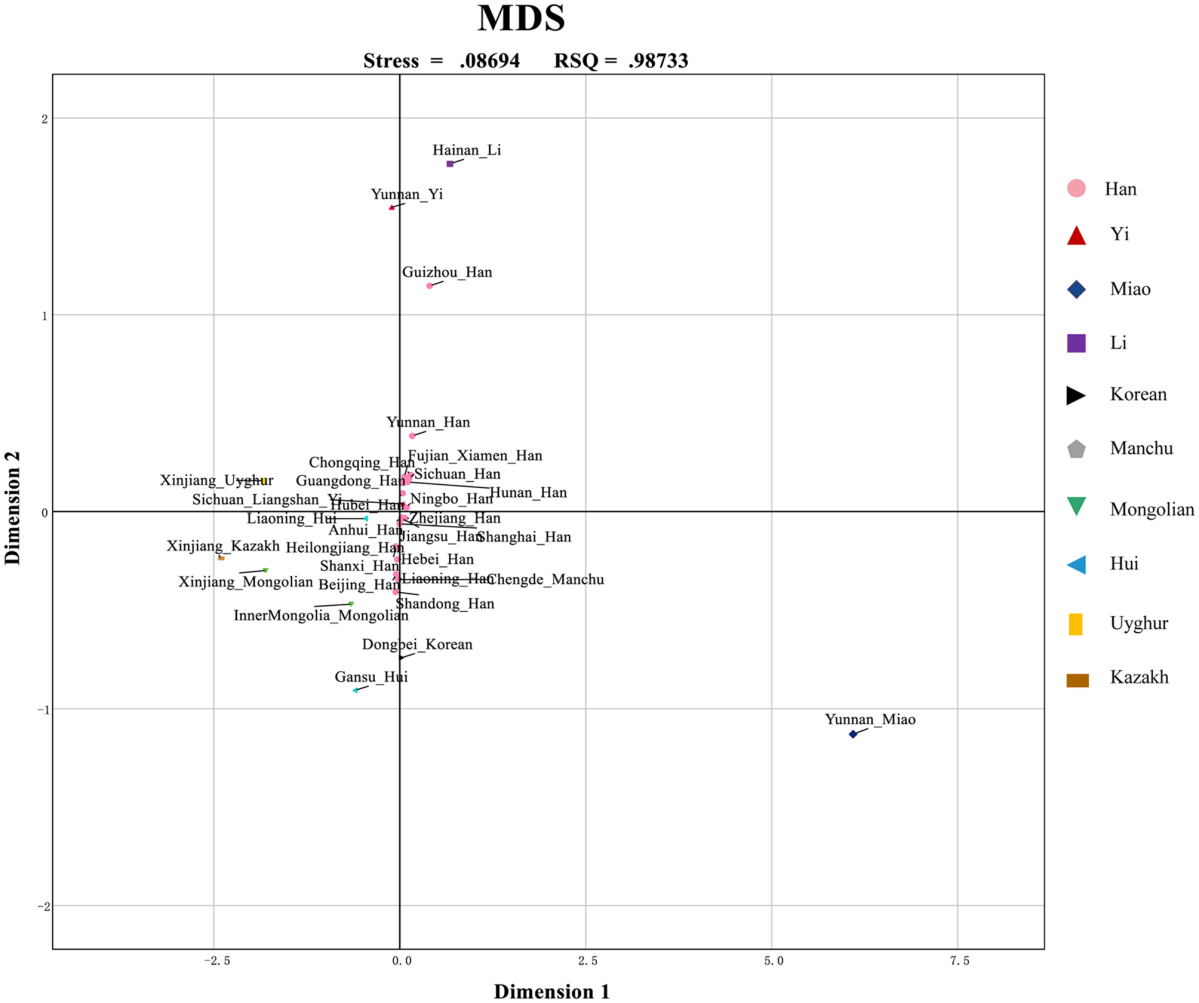
Multidimensional scaling (MDS) plots displaying the genetic relationships between the Yi ethnic minority and 30 reference populations.

**Figure 3:**
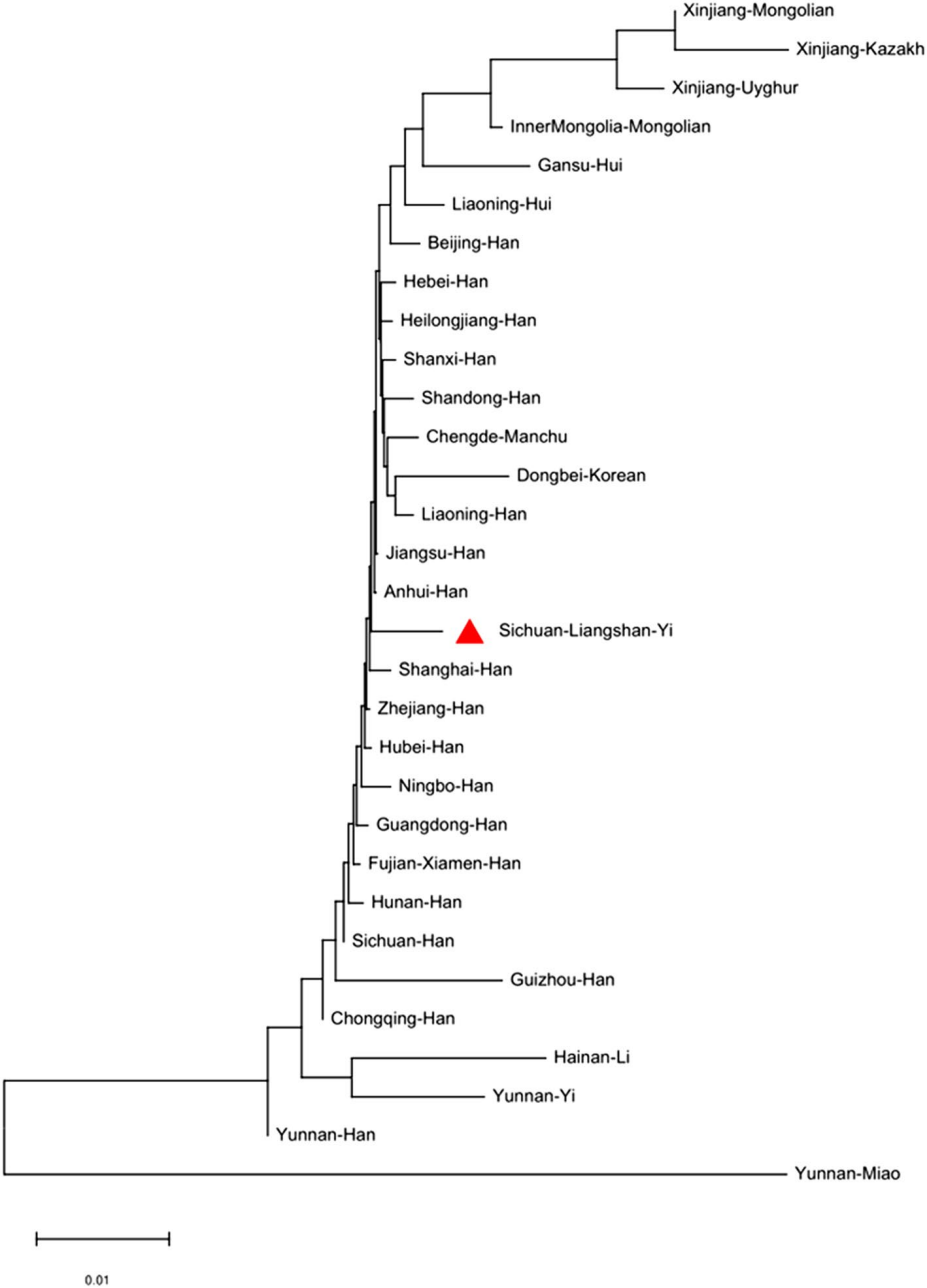
Phylogenetic tree displaying the genetic relationships between the population and 30 reference populations. The phylogenetic tree was constructed using the neighbour-joining method based on 19 overlapping STR loci with MEGA-X software.

Nei’s standard genetic distance matrix among 31 populations (Fig. 1, Supplementary Table S3) is presented in Table 2. From Table 2, we found that the nearest genetic distance to the Yi population was the Sichuan Han (0.0056), followed by the Anhui Han (0.0058) and the Guangdong Han (0.0059). The Euclidean distance model of a multidimensional scale (MDS) and a representation plot are indicated in Fig. 2. Eighteen Han origin groups (except Guizhou Han), Chengde Manchu and Liaoning Hui are closely located together and stand far apart from Yunnan Miao, Xinjiang Kazakh, Xinjiang Uyghur, Gansu Hui, Xinjiang Mongolian, Inner Mongolia Mongolian, Dongbei Korean, Yunnan Yi and Hainan Li ethnic populations. The neighbour joining (NJ) phylogenetic tree (Fig. 3) of Nei’s standard distance matrix showed that Yunnan Han, Sichuan Han, Guizhou Han, Chongqing Han, Hunan Han, Fujian Xiamen Han, Guangdong Han, Zhejiang Han, Ningbo Han, Hubei Han, Shanghai Han, Anhui Han, and Jiangsu Han, are formed as a southern China Han population. Hebei Han, Heilongjiang Han, Shanxi Han, Shandong Han, Liaoning Han, and Beijing Han are formed as a northern China Han population. Xinjiang Uyghur, Xinjiang Mongolian, Xinjiang Kazakh and Inner Mongolia Mongolian ethnic minorities presented a relatively close but distant relationship to the Han groups. Chengde Manchu, Dongbei Korean, Gansu Hui, and Liaoning Hui ethnic minorities were relatively close to the Northern China Han population. The Yunnan Han and Yunnan Miao ethnic minorities clustered a unique cluster and were relatively obviously distant from the Southwest China Han population. The Yunnan Yi and Hainan Li ethnic minorities clustered a unique cluster. Yunnan Miao showed an obvious distant relationship from the other groups. Thus, the NJ phylogenetic tree and MDS corresponded with the cultural, historical, and geographical distribution of the studied majority groups.

**Table 2:** Genetic distances between the Liangshan Yi ethnic minority and other 30 relative Chinese reference populations.

Populations	[01]	[02]	[03]	[04]	[05]	[06]	[07]	[08]	[09]	[10]	[11]	[12]	[13]	[14]	[15]	[16]
[01] Sichuan-Liangshan-Yi																
[02] Hainan-Li	0.025750															
[03] Xinjiang-Mongolian	0.028549	0.050866														
[04] Yunnan-Yi	0.018894	0.024503	0.039871													
[05] Gansu-Hui	0.014979	0.040329	0.025844	0.033464												
[06] Dongbei-Korean	0.015962	0.031975	0.028352	0.029496	0.021855											
[07] Yunnan-Miao	0.092912	0.095559	0.119075	0.100579	0.106409	0.094044										
[08] Liaoning-Hui	0.009422	0.029400	0.018967	0.021640	0.013865	0.014438	0.099201									
[09] Chengde-Manchu	0.007859	0.030839	0.022603	0.025357	0.013535	0.011137	0.091966	0.008440								
[10] InnerMongolia-Mongolian	0.015378	0.039511	0.011868	0.028261	0.015353	0.016794	0.103780	0.010714	0.011859							
[11] Xinjiang-Kazakh	0.037212	0.058550	0.007614	0.045343	0.029876	0.037858	0.142544	0.026298	0.034541	0.019085						
[12] Xinjiang-Uyghur	0.025872	0.045085	0.011835	0.034411	0.026305	0.029803	0.124435	0.019745	0.025972	0.016839	0.011517					
[13] Sichuan-Han	0.005648	0.018020	0.026896	0.015056	0.014144	0.013020	0.077348	0.007412	0.005995	0.013076	0.036483	0.025095				
[14] Chongqing-Han	0.007721	0.018484	0.027253	0.016854	0.014515	0.014854	0.078627	0.008823	0.007035	0.014456	0.037653	0.027587	0.002078			
[15] Guizhou-Han	0.020614	0.031819	0.039213	0.026460	0.030001	0.025781	0.094031	0.019618	0.021886	0.027349	0.047816	0.034604	0.014004	0.014588		
[16] Yunnan-Han	0.006906	0.016794	0.028324	0.010822	0.018298	0.014944	0.074722	0.008705	0.008433	0.015455	0.037965	0.025986	0.001788	0.003169	0.014158	
[17] Guangdong-Han	0.005922	0.018861	0.022993	0.014744	0.013615	0.011778	0.084187	0.005894	0.005950	0.011967	0.031429	0.022430	0.001641	0.002900	0.013522	0.002648
[18] Fujian-Xiamen-Han	0.006453	0.018385	0.026001	0.016991	0.014463	0.012037	0.085105	0.007089	0.005277	0.013563	0.035306	0.024422	0.001195	0.002559	0.014201	0.002466
[19] Zhejiang-Han	0.006496	0.022397	0.024301	0.019069	0.012438	0.011066	0.081691	0.005859	0.004011	0.010919	0.033828	0.024319	0.001416	0.002725	0.014448	0.003367
[20] Ningbo-Han	0.008409	0.022860	0.025295	0.018791	0.015876	0.012809	0.083613	0.007885	0.005971	0.013571	0.034989	0.026343	0.002973	0.004819	0.016046	0.004815
[21] Jiangsu-Han	0.006092	0.024332	0.022755	0.020946	0.011339	0.009789	0.084965	0.005739	0.003293	0.009965	0.032692	0.023651	0.001937	0.003297	0.014903	0.004470
[22] Shanghai-Han	0.006892	0.024981	0.022654	0.020288	0.012748	0.012261	0.090540	0.007062	0.004817	0.009349	0.031768	0.022457	0.002975	0.004067	0.017008	0.005042
[23] Anhui-Han	0.005819	0.024700	0.022603	0.020045	0.012074	0.009811	0.085803	0.005621	0.003051	0.010307	0.032543	0.023065	0.002072	0.003504	0.014674	0.004266
[24] Hubei-Han	0.006176	0.021054	0.023887	0.017491	0.013408	0.011675	0.084161	0.006301	0.004348	0.011268	0.033296	0.022926	0.001397	0.002896	0.013242	0.002869
[25] Hunan-Han	0.006794	0.019772	0.026274	0.017298	0.015606	0.014265	0.081781	0.008206	0.006735	0.013825	0.037187	0.026150	0.001931	0.003249	0.014437	0.003415
[26] Beijing-Han	0.008805	0.029539	0.020546	0.023514	0.011482	0.012816	0.092097	0.007222	0.005867	0.009506	0.030207	0.023904	0.005687	0.006513	0.019822	0.008162
[27] Hebei-Han	0.007631	0.029126	0.021670	0.021931	0.012942	0.011147	0.092269	0.006019	0.003609	0.009642	0.031024	0.022725	0.003833	0.005224	0.017134	0.006631
[28] Shandong-Han	0.008115	0.032953	0.021179	0.026308	0.013235	0.011778	0.088427	0.009304	0.004474	0.010120	0.031735	0.023591	0.005974	0.007568	0.018972	0.008244
[29] Heilongjiang-Han	0.007180	0.027861	0.021029	0.023461	0.011980	0.009883	0.087543	0.006161	0.003672	0.009851	0.031539	0.022568	0.003464	0.004738	0.016383	0.006449
[30] Liaoning-Han	0.008060	0.031168	0.021866	0.025757	0.012781	0.009806	0.092921	0.007589	0.004344	0.009513	0.031961	0.024941	0.005461	0.006813	0.019937	0.008773
[31] Shanxi-Han	0.007295	0.028456	0.019978	0.021881	0.012450	0.009744	0.085970	0.006169	0.003669	0.009214	0.029500	0.021817	0.003710	0.005243	0.017063	0.006226

Populations	[17]	[18]	[19]	[20]	[21]	[22]	[23]	[24]	[25]	[26]	[27]	[28]	[29]	[30]	[31]	
[01] Sichuan-Liangshan-Yi																
[02] Hainan-Li																
[03] Xinjiang-Mongolian																
[04] Yunnan-Yi																
[05] Gansu-Hui																
[06] Dongbei-Korean																
[07] Yunnan-Miao																
[08] Liaoning-Hui																
[09] Chengde-Manchu																
[10] InnerMongolia-Mongolian																
[11] Xinjiang-Kazakh																
[12] Xinjiang-Uyghur																
[13] Sichuan-Han																
[14] Chongqing-Han																
[15] Guizhou-Han																
[16] Yunnan-Han																
[17] Guangdong-Han																
[18] Fujian-Xiamen-Han	0.001870															
[19] Zhejiang-Han	0.001941	0.001196														
[20] Ningbo-Han	0.003496	0.003138	0.002753													
[21] Jiangsu-Han	0.002244	0.001744	0.000614	0.002752												
[22] Shanghai-Han	0.003194	0.002735	0.002107	0.004891	0.002296											
[23] Anhui-Han	0.002228	0.001721	0.000748	0.002610	0.000408	0.002215										
[24] Hubei-Han	0.001869	0.001044	0.000854	0.003096	0.001178	0.002167	0.001118									
[25] Hunan-Han	0.002586	0.002188	0.002719	0.004191	0.002918	0.003393	0.002808	0.002396								
[26] Beijing-Han	0.004897	0.006246	0.004309	0.007354	0.003418	0.004983	0.003515	0.004753	0.005854							
[27] Hebei-Han	0.003836	0.003495	0.002108	0.004589	0.001393	0.003030	0.001437	0.002516	0.004755	0.004409						
[28] Shandong-Han	0.005755	0.005875	0.004138	0.006391	0.002802	0.005345	0.003026	0.004100	0.006781	0.004826	0.003393					
[29] Heilongjiang-Han	0.003625	0.003468	0.001936	0.004239	0.001206	0.003303	0.001390	0.002654	0.004500	0.004056	0.001905	0.003145				
[30] Liaoning-Han	0.004927	0.005372	0.003710	0.005942	0.002821	0.004619	0.003021	0.004514	0.006942	0.005912	0.002885	0.004206	0.002793			
[31] Shanxi-Han	0.003482	0.003646	0.002314	0.003844	0.001612	0.003050	0.001629	0.002700	0.004832	0.004140	0.002094	0.003253	0.002055	0.003218

The *F*_*st*_ values and corresponding p values of the population comparisons between the Liangshan Yi population and the other 30 compared populations at 19 STR loci are shown in Supplementary Table S4. Yunnan Miao showed significant genetic differences with Liangshan Yi at seventeen STR loci and no significant differences were observed with the exception of Yunnan Miao at the locus of D5S818 and Penta D. The calculation revealed that there were statistically significant differences between Liangshan Yi and Xinjiang Mongolian at twelve STR loci, followed by Xinjiang Kazakh at nine STR loci, Yunnan Yi at six STR loci, Hainan Li at five STR loci, Zhejiang Han, Yunnan Han and Fujian Xiamen Han at two STR loci, and among the Dongbei Korean, Xinjiang Uyghur, Sichuan Han, Guizhou Han, Guangdong Han, Ningbo Han, Jiangsu Han, Anhui Han, Hubei Han, Hebei Han, Shanxi Han and Heilongjiang Han at one locus after Bonferroni correction (p < 0.0003). However, no statistically significant differentiation was obtained for the Gansu Hui, Liaoning Hui, Chengde Manchu, Inner Mongolia Mongolian, Chongqing Han, Shanghai Han, Hunan Han, Beijing Han, Shandong Han or Liaoning Han at any of the 19 STR loci.

## Discussion

China is currently populated by 1,443,497,378 people who belong to at least 56 officially recognized linguistically and ethnically different Chinese groups, according to China’s Seventh National Census Bulletin (http://www.stats.gov.cn/tjsj/tjgb/rkpcgb/) published on May 11, 2021. Compared with the China’s Sixth National Census in 2010, the Han population increased by 60,378,693 (4.93%) and the ethnic minorities increased by 11,675,179 (10.26%) (http://www.stats.gov.cn/tjsj/tjgb/rkpcgb/qgrkpcgb/202106/t20210628_1818821.html), showing a more than twofold increase for ethnic minorities. Genetic studies of Chinese populations from ethnic minorities are of great interest due to China’s complex demographics, complex geographical characteristics and large population sizes. The Liangshan Yi Autonomous Prefecture (LYAP) in Sichuan Province has the single largest Yi community. However, the genetic polymorphisms responsible for the autosomal STR loci of the Yi minority in LYAP are unclear, and the genetic relationships between the Yi minority population and other ethnic populations are unknown.

In this study, by recruiting 1016 unrelated individuals, the total discrimination power (TDP) and the cumulative probability of exclusion (CPE) for 19 STRs were found to be 0.999999999999999999999897 and 0.9999999597, respectively. Totals of 273 alleles for 19 STRs and 8–22 alleles for each locus were found. The observed heterozygosity (H_obs_) was 78.44%, the expected heterozygosity (H_exp_) was 79.89%, the discrimination power (DP) was 92.66%, and the probability of exclusion (PE) was 57.68%. Penta E presented the highest levels of H_obs_ (0.8917) and DP (0.9839), whereas TPOX showed the lowest H_obs_ of 0.6230 and DP of 0.8154. Nei’s standard genetic distance matrix among 31 populations found that the nearest genetic distance to the Yi population was the Sichuan Han (0.0056), followed by the Anhui Han (0.0058) and Guangdong Han (0.0059). Nei’s standard genetic distance of the Yi population in Sichuan Province to the Yi population in Yunnan Province was 0.0189, showing the 24th nearest genetic distance. Surprisingly, Yunnan Miao showed an obvious distant relationship from other groups, which was consistent with other analyses^11^, showing far distance to Filipinos from the Philippines. Altogether, the NJ phylogenetic tree and MDS corresponded with the cultural, historical, and geographical distribution of the studied majority groups.

The *F*_*st*_ values and corresponding p values of the population comparisons between the Liangshan Yi population and the other 30 compared populations indicated that no statistically significant differentiation was obtained with Gansu Hui, Liaoning Hui, Chengde Manchu, Inner Mongolia Mongolian, Chongqing Han, Shanghai Han, Hunan Han, Beijing Han, Shandong Han or Liaoning Han at any of the 19 STR loci. The exploration of the origin and diversification of ethnic populations may be affected by the natural environment, cultural background, population migration and national policies.

In conclusion, our work was the first to report the forensic parameters and allele frequencies of 19 autosomal STR markers of the Yi group in Liangshan, China, and the population genetic relationships between the Yi minority and 30 other neighbouring populations. These 19 STR makers could provide highly informative polymorphisms for individual identification, paternity testing and genetic population analyses.

## Methods

### Sample collections and genomic DNA isolation

Bloods from 1016 unrelated healthy Yi individuals were recruited from Xichang City and its surrounding areas of the LYAP, Sichuan Province, P. R. China. Chelex-100 protocol was used to extract DNA^12,13^. The study was approved by the institutional review boards of Southwest Medical University. Informed consent from participants and ethical approval from the Committee of Southwest Medical University were obtained. All experiments were performed in accordance with relevant guidelines and regulations.

### PCR amplification and STR genotyping

In this study, the Goldeneye™ DNA Identification System 20A Kit from Beijing in China was used, which included 19 autosomal STRs, namely D2S1338, D3S1358, D5S818, D6S1043, D7S820, D8S1179, D12S391, D13S317, D16S539, D18S51, D19S433, D21S11, CSF1PO, Penta D, vWA, TPOX, Penta E, TH01, and FGA.

PCRs were generated according to the manufacturer's instructions. For details, a 10 μL PCR volume was used for each sample, including 2.5 × PCR Buffer III 4.0 μL, 5 × 20A Primer Mix 2.0 μL, Taq DNA Polymerase III 0.16 μL and ddH_2_O 3.84 μL. PCR amplification was conducted using the Goldeneye™ DNA ID System 20A Kit from Peoplespot (Cat#: 20AMC401, Beijing, China) in an Applied Biosystems Veriti® 96-Well Thermal Cycler (Applied Biosystems, Life Technology, USA) with the following steps: pre-denatured 96 °C for 2 min, followed by 30 cycles of 94 °C for 5 s and 60 °C for 70 s, then a step of 60 °C for 30 min, and finally held at 15 °C until for capillary electrophoresis. The PCR products were genotyped by using the 3500 Dx Genetic Analyzer (Applied Biosystems, Life Technology, USA) and analyzed by GeneMapper ID-X software (Thermo Fisher, USA)^14^. For quality, DNA as a control was also supplied in this kit. Genotype analysis for STR was performed by reference to the provisions of the ISO/IEC 17025:2005 General Requirements for the Competence of Testing, the Specification for Parentage Testing by China (GB/T 37223-2018).

### Data analysis

Modified-PowerStats software was used to evaluate forensic parameters, allele frequencies and Hardy–Weinberg equilibrium (HWE). The Arlequin v3.5 software was used to analyze linkage disequilibrium (LD) and population differentiation between the studied group and previously published data^15^. Nineteen STR information from 30 Chinese populations, including one Manchu^16^, two Hui^17,18^, one Kazakh^19^, one Uyghur^20^, one Miao^21^, one Yi^3^, one Li^11^, one Korean^22^, two Mongolian^23,24^ and 19 Han^25–34^ populations (Fig. 1), were extracted from other studies to evaluate the genetic similarities and divergences. The longitude and latitude of all 31 reference populations are presented in Supplementary Table S3. A modified PHYLIP program was used to calculate Nei’s standard genetic distance between Yi populations and the 30 other populations reported in previous studies^35^. MEGA-X software was used to develop and visualize the phylogenetic tree^36^.

### Compliance with ethical standards

This study was approved by Southwest Medical University (KY2021168).

## Supplementary Information

Supplementary Table S1.

Supplementary Table S2.

Supplementary Table S3.

Supplementary Table S4.

## Data Availability

All data used for the analyses in this report are available from the corresponding author on reasonable request.
